# A Novel Method for Rapid Screening of Salmonidae Ingredients and Accurate Detection of Atlantic Salmon (*Salmo salar*) Simultaneously Using Duplex Real-Time PCR Coupled with Melting Curve Analysis

**DOI:** 10.3390/molecules29204904

**Published:** 2024-10-16

**Authors:** Shihui Wang, Xiong Xiong, Hongwei Song, Tianlong Wang, Yi Li, Libin Wang

**Affiliations:** 1 College of Food Science and Light Industry, Nanjing Tech University, Nanjing 211816, China; 2 College of Light Industry and Food Engineering, Nanjing Forestry University, Nanjing 210037, China

**Keywords:** *Salmo salar*, Salmonidae, real-time PCR, melting curve analysis, duplex PCR

## Abstract

The substitution of ingredients with Salmonidae, particularly *Salmo salar*, has led to widespread reports of financial losses and health risks globally, emphasizing the urgent need for the development of a rapid and precise method for species identification. The aim of the present study was to develop a novel method for the rapid screening of Salmonidae ingredients and the accurate detection of *S. salar* simultaneously using multiplex real-time PCR coupled with melting curve analysis. Specifically, primer sets specific for *S. salar* and Salmonidae were cross-confirmed. Moreover, the reaction system and conditions of a real-time duplex PCR were optimized, and the proposed methodology was verified, proving that the assay has good specificity and sensitivity. Clear and distinguishable melting peaks, with expected Tm values of around 80 °C (*S. salar*) and 84 °C (Salmonidae), were observed for twelve products, proving the presence of *S. salar*. However, four products were not derived from *S. salar*, but they could have belonged to another species within the Salmonidae family due to the presence of only one specific melting peak at a Tm value of about 84 °C. Therefore, the novel assay in the present study allows for the fast and accurate screening of Salmonidae ingredients and the detection of *S. salar* simultaneously.

## 1. Introduction

Salmonidae falls under the order Salmoniformes and encompasses numerous commercially significant species, including wild-caught varieties like *Oncorhynchus tshawytscha*, *O. nerka*, *O. kisutch*, *O. keta*, and *O. gorbuscha*, as well as the farmed species of *Salmo salar* and *O. mykiss*. Salmonidae is a critical contributor to global fish production, accounting for a total output of 4.85 million tons in 2022, with 82% of this production coming from aquaculture [1]. In recent years, the global demand for Salmonidae species has surged, fueled by their nutritional value, culinary versatility, and an increasing consumer preference for sustainable and healthy food options [2,3]. In particular, *S. salar* represents one of the most esteemed salmonid species owing to its status as an exceptional source of high-quality protein and essential micronutrients vital for balanced nutrition and optimal health [1,4]. For consumption, raw *S. salar*, renowned for its smooth taste and vibrant color, is typically sliced into fillets and enjoyed directly as sashimi [5]. Furthermore, *S. salar* can also be marketed in various forms, including smoked fillets, dry-dressed fillets, and even as a powdered supplement incorporated into baby food [6,7].

However, the increasing popularity of these Salmonidae products has also led to an escalation in the complexity of supply chains, posing challenges in ensuring food safety, authenticity, and traceability [8,9]. Specifically, a previous study revealed the presence of only chicken and pork in Salmonidae-claimed fish products in China [10]. Moreover, *S. salar* is frequently adulterated with closely related species, for instance, *O. mykiss* [11], likely due to the large price gap between *O. mykiss* (about RMB 20/kg, or USD 3/kg) and *S. salar* (about RMB 110/kg or USD 16/kg) (obtained from one of the most popular online markets in China between 3 April and 10 May 2024). This situation could become even worse with the widespread use of unacceptable market names in some countries [12,13]. To this end, the accurate identification of Salmonidae ingredients, particularly the discrimination of *S. salar* from other species, is crucial for regulatory compliance, protecting consumer health, and maintaining market integrity.

Traditional approaches to species identification, which rely on morphological analysis and biochemical tests, are frequently time consuming, labor intensive, and susceptible to inaccuracies, especially when examining processed or mixed products. To address these shortcomings, multiple protein-centric methodologies (encompassing electrophoretic, chromatographic, and immunological techniques) have been devised [14]. These methods are generally trustworthy for identifying species in fresh or minimally processed foods but may lose practicality with heavily processed items [15]. As an alternative, DNA-based strategies have emerged as potent instruments for swift and precise species identification. Notably, various DNA amplification techniques have been developed in recent years, including the polymerase chain reaction (PCR), the denaturation bubble-mediated strand exchange amplification (SEA), and loop-mediated isothermal amplification (LAMP) [16,17]. Among these, PCR stands out as the most prevalent technique, with a diverse array of PCR-based diagnostic methods having been successfully employed for species identification across diverse fish families [18,19,20].

Of these PCR-based methods, multiplex PCR has emerged as one of the most pragmatic techniques owing to its swiftness, ease of execution, and capability to simultaneously detect multiple species in a single reaction [21,22]. Specifically, multiplex PCR utilizes distinct primer sets to amplify characteristic fragments of varying sizes for each species group, enabling rapid differentiation between groups through the distinctive banding patterns observed during agarose gel electrophoresis [23,24]. Additionally, multiplex PCR methods can also be enhanced with real-time protocols using probes or the cost-effective SYBR Green I dye for validation [25,26]. Recently, advancements have been made regarding utilizing SYBR Green I real-time multiplex PCR coupled with melting curve analysis for accurately identifying various species, including three Mytilus species [27], eleven meat species [28], seven Lophius species [29], and eleven crab species [30].

In this study, we present a novel method for the rapid screening of Salmonidae ingredients and the accurate detection of *S. salar* simultaneously using a duplex real-time PCR system combined with melting curve analysis. By employing multiple primers targeting specific genetic signatures, our approach enables the concurrent detection of *S. salar* and the presence of any Salmonidae component in a sample, thereby simplifying the identification process. This novel method addresses an urgent requirement in the seafood industry by providing a rapid, reliable, and economical solution for species identification.

## 2. Results and Discussion

### 2.1. Optimization of the Duplex Real-Time PCR Assay

To develop a melting curve-based duplex PCR assay for the rapid screening of Salmonidae ingredients and the accurate detection of *S. salar* simultaneously in one tube, two primer sets specific for Salmonidae and *S. salar* were designed and obtained from our previous studies (Table 1). The specificity of each primer set was preferentially confirmed through single PCR with genomic DNA of various fish species. A *S. salar* species-specific primer set (SalmonF-1/SalmonR-1) amplified only the target species, but not other nontarget species. The universal Salmonidae-specific primer (FCYTB346/RCYTB530) targeting the Cytb gene amplified a specific PCR product for all of the Salmonidae samples used (Figure S1), as expected.

To prove the specificity of the assay using different equipment and in different situations, the reaction was also performed in a final volume of 20 µL containing PCR reagents that were similar to the ones described in Section 2.3 (PCR amplification and sequencing). The amplification was performed on a LightCycler^®^ 96 real-time PCR system (Roche, Basel, Switzerland), and the results highlighted the same specificity for each primer set.

**Figure 1:**
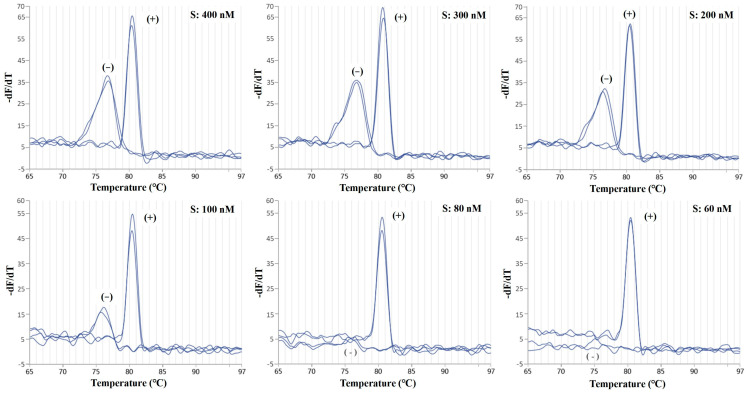
Melting curve analysis of the real-time PCR assay using the primers specific for *S. salar*. Primers specific for *S. salar* (coded as S) were studied, with the concentration ranging from 60 nM to 400 nM. (+) represents *S. salar*; (−) represents the negative control.

During the development and optimization of the duplex PCR assay, PCR-related factors such as the concentrations of Taq DNA polymerase, PCR buffer, dNTPs, and the primer set were adjusted to minimize nonspecific interactions [26]. In particular, the proportion of primers strongly impacts the duplex PCR reaction: adding the same concentration of primers may lead to the mutual inhibition of primers, resulting in the loss of a species signal [33]. By changing the concentration of various primers in the PCR amplification reaction system, the specific amplification of different species can be discovered. In the present study, we first gradually increased the *S. salar* species-specific primer sets (60, 80, 100, 200, 300, and 400 nM) to observe the impact of primer quantity on the real-time single PCR reaction. The results highlighted a significant nonspecific amplification when the *S. salar* species-specific primer set was at a concentration greater than 80 nM (Figure 1).

**Figure 2:**
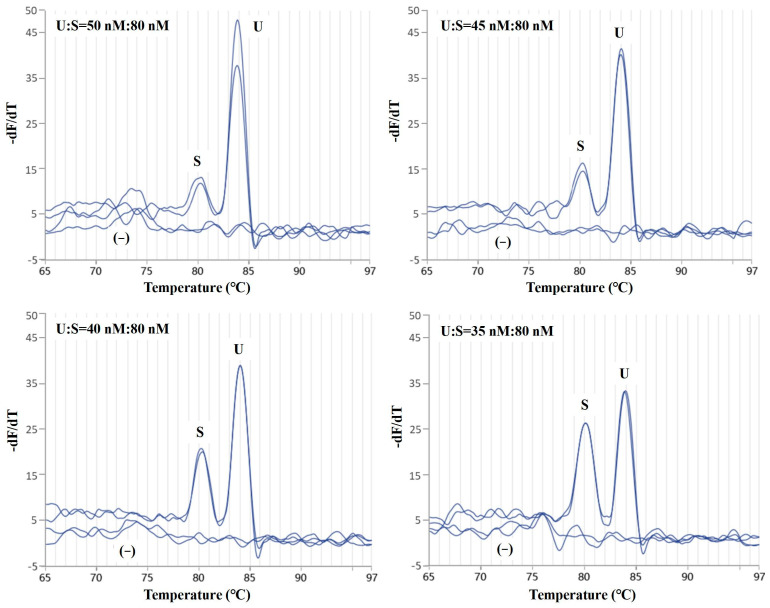
Melting curve analysis of the duplex real-time PCR assay using the universal primers and primers specific for *S. salar*. Universal primers for Salmonidae (coded as U) were studied, with the concentration ranging from 35 nM to 50 nM. Primers specific for *S. salar* were coded as S; (−) represents the negative control.

Based on *S. salar* species-specific primer sets at a concentration of 80 nM in the reaction system, the results of duplex PCR amplification are shown in Figure 2. The initial concentration of the Salmonidae-specific primer sets was 50 nM, and the final concentrations of the primers were decreased to 45, 40, and 35 nM. Melting curve analysis after real-time PCR produced similar efficiency, as 35 nM and 80 nM of the *S. salar* species-specific primers and the Salmonidae-specific primers were added, respectively. In this case, there were two specific peaks at Tm 80.6 ± 0.4 °C and 83.8 ± 0.2 °C, which were generated from the PCR products amplified by the *S. salar* species-specific primers and the Salmonidae-specific primers (Figure 2). A gap of 3 degrees clearly differentiates the Salmonidae group from the *S. salar* species. With the optimal primer concentration, the duplex PCR method was successfully established, and it could effectively amplify the genes of *S. salar* and Salmonidae.

### 2.2. Evaluation of Sensitivity and Specificity

**Figure 3:**
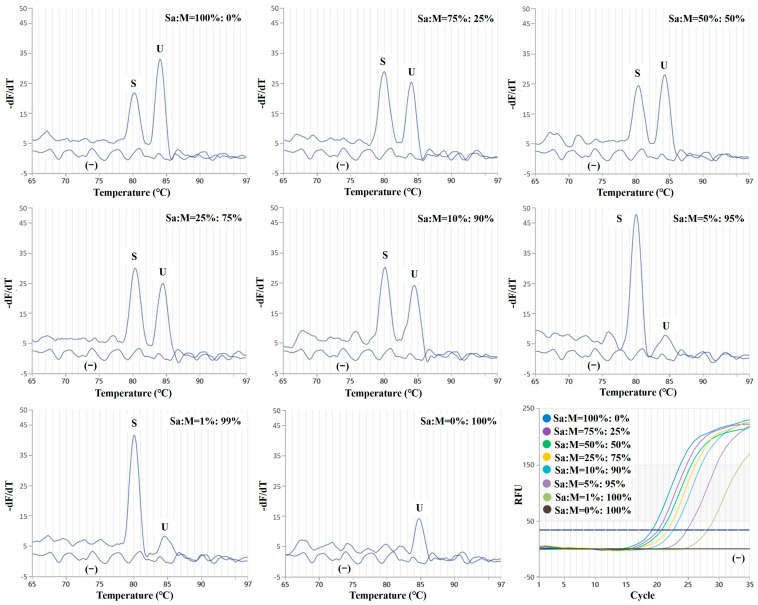
Evaluation of the duplex real-time PCR assay regarding simulated tissue mixtures. The addition of increasing amounts of *S. salar* tissue to *O. masou* mixtures was simulated; *S. salar* tissue was coded as S; *O. masou* tissue was coded as M. Primers specific for *S. salar* were coded as S; universal primers for Salmonidae were coded as U; (−) represents the negative control.

To validate the specificity and effectiveness of the established duplex real-time PCR assay, we conducted a study utilizing binary mixtures of raw tissue containing varying proportions of *S. salar* and *O. masou* (Figure 3). The objective of this experiment was to evaluate the system’s capacity to precisely identify and amplify target DNA from combined samples, mirroring real-life situations where distinguishing between species is crucial. Notably, the duplex real-time PCR assay only yielded a low melting peak (84.1 °C) in the 0% *S. salar* and 100% *O. masou* binary mixtures, effectively confirming the absence of false positives and negligible cross-reactivity with *O. masou* tissue. The most compelling validation occurred when the binary mixtures contained a minimum amount of *S. salar* tissue, specifically at the 1% *S. salar* concentration (Figure 3). A Salmonidae-specific melting peak can be observed for all the mixed samples, indicating that this method can effectively detect adulterated Salmonidae samples.

**Figure 4:**
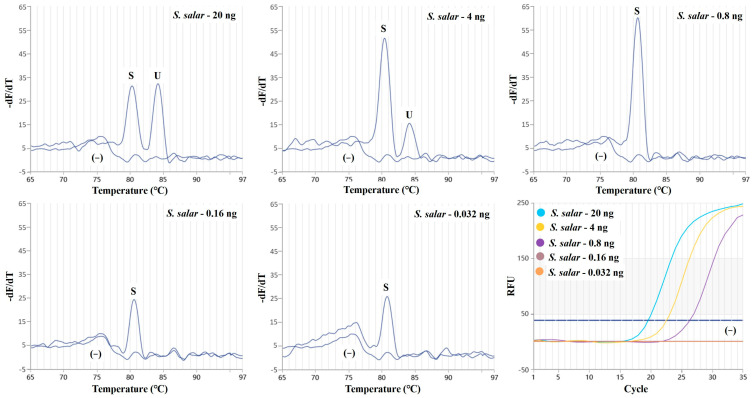
Sensitivity evaluation of the duplex real-time PCR assay. The 5-fold serial dilutions of *S. salar* DNA (from 20 ng to 0.032 ng) were prepared. Primers specific for *S. salar* were coded as S; universal primers for Salmonidae were coded as U; (−) represents the negative control.

To determine the absolute limit of detection, a series of 5-fold dilutions of the *S. salar* DNA template (20 ng, 4 ng, 0.8 ng, 0.16 ng, and 0.032 ng) was carried out to test the performance of this duplex real-time PCR method. The sensitivity was determined on the basis of the minimum amount of DNA that could produce a detectable signal. The result showed that the lowest quantity of detectable DNA was 0.032 ng for *S. salar* and 4 ng for Salmonidae (Figure 4). The main reason for the poor sensitivity in the present study could be attributed to a competition between the two primer sets. Also, to avoid a nonspecific signal, the primer concentration of the present study was lower than that of other similar studies in which the primer concentration was generally 300–500 nM [21,33].

**Figure 5:**
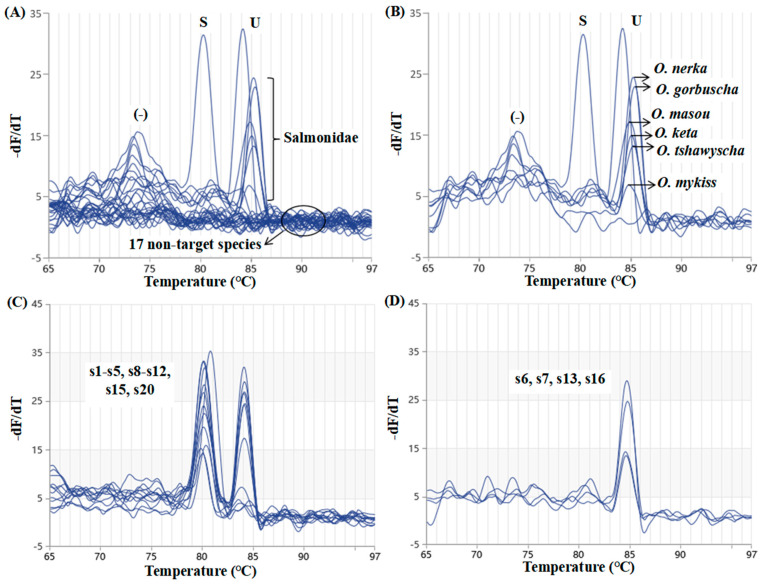
Specificity evaluation of the duplex real-time PCR (**A**,**B**) and its validation using commercial products (**C**,**D**). Primers specific for *S. salar* were coded as S; universal primers for Salmonidae were coded as U; (−) represents the negative control. s1–s20 were the commercial products described in Table 2.

Although the specificity of each primer set in the simple PCR system was established, it was necessary to analyze the specificity in the duplex PCR system to avoid the effects of the interference caused by the interaction of multiple primers on the specificity that was observed in the single reaction system. The specificity of the duplex real-time PCR assay was further verified with the genomic DNA of 6 closely related salmonid species and other 17 commercially important fish species commonly encountered in foodstuffs (belonging to Pleuronectiformes, Gadiformes, Perciformes, Clupeiformes, Scorpaeniformes, and Cypriniformes). As shown in Figure 5A,B, each targeted region was specifically amplified using the predicted Tm of the PCR product of each specific primer set (*S. salar* species-specific primer sets, 80.6 ± 0.4 °C; Salmonidae-specific primer sets, 83.8–84.9 °C). Moreover, the melting peak was also highlighted for each Salmonidae species in Figure 5B, namely 83.8 °C for *O. masou*, 84.1 °C for *O. keta*, 84.1 °C for *O. mykiss*, 84.4 °C for *O. tshawytscha*, 84.8 °C for *O. nerka*, and 84.9 °C for *O. gorbuscha*. All the other 17 nontarget species failed the amplification.

### 2.3. Method Validation Using Commercial Products

The application of the duplex real-time PCR method developed in the present study to commercial products was verified in Figure 5C,D and Table 2. Clear and distinguishable melting peaks with expected Tm values of around 80 °C and 84 °C for s1–5, s8–12, s15, and s20 were observed (Figure 5C). In other words, these 12 products were successfully identified as *S. salar*, proving that these products had the correct species label. However, there was only one specific melting peak at a Tm value of about 84 °C for s6, s7, s13, and s16. Therefore, these products were not derived from *S. salar*, but they could belong to another species within the Salmonidae family. Finally, no melting peak was observed for the remaining four products (s14 and s17–19), and thus, no Salmonidae species were identified, highlighting a high probability of mislabeling.

Finally, DNA barcoding was also used to identity the species of these commercial products. The results indicated that the samples that produced two specific melting peaks, namely, s1–5, s8–12, s15, and s20, were successfully identified as *S. salar* (Table 2), demonstrating the feasibility of the real-time duplex PCR assay with melting curve analysis developed in the present study. All four products with only one specific melting peak were identified as *O. mykiss*, *O.gorbuscha*, and *O. keta*. The four remaining products without melting peaks also failed during DNA barcoding. We consider several possible reasons for this: (1) DNA degradation caused the amplification of 313 bp to fail during DNA barcoding, and (2) some inhibitors during fish processing were introduced [34].

## 3. Materials and Methods

### 3.1. Sample Collection

Reference samples comprised of fresh or frozen specimens from 24 genuine fish species, specifically *O. mykiss*, *O. keta*, *O. masou*, *O. nerka*, *O. gorbuscha*, *O. tshawytscha*, *S. salar*, *Gadus chalcogrammus*, *Melanogrammus aeglefinus*, *Lateolabrax japonicus*, *Epinephelus costae*, *Pennahia argentata*, *Upeneus japonicas*, *Mullus barbatus*, *Lates niloticus*, *Xiphias gladius*, *Sardina pilchardus*, *Polydactylus sextarius*, *Odontobutis potamophila*, *Anoplopoma fimbria*, *Thunnus obesus*, *Pleuronectes platessa*, *Cyprinus carpio*, and *Thunnus albacores*. These species were sourced from our previous studies [31,32,34], official food control authorities, and local supermarkets in Nanjing, China, and were morphologically verified by experts in fish taxonomy. Additionally, the authenticity of all fish samples was doubly confirmed through DNA barcoding [35], and the sequences deposited in Genbank (accession numbers MN850428, MN893169, MN850430-MN850436, MN843687, MN893177, MN879569, MN893179, MN893180, MN893181, MN893182, MN893187, MN879570, MN843731, MN893197, MN893173, MN893185, MN893174, and MK843655).

Furthermore, for method validation, a total of 20 commercial fish products (including dried and frozen varieties) were randomly procured from local markets in Nanjing, China (Table 2). Label inspection revealed that all products were merely labeled with the Chinese common name “San Wen Yu”, which, based on our previous investigation [13], suggests potential Salmonidae without specifying a particular species. Upon arrival at the laboratory, a visual examination of the product contents was conducted, followed by washing with distilled water. All samples were then stored at −20 °C for subsequent analysis.

### 3.2. DNA Extraction

The extraction of total DNA from all individual samples was accomplished utilizing the TIANamp Genomic DNA Kit (Tiangen, Beijing, China), according to the protocol provided by the manufacturer. In summary, 50 mg of flesh from each individual was mixed with 200 µL of GA lysis buffer and 20 µL of Proteinase K (20 mg/mL). This mixture was then incubated at 56 °C for 60 min. Following this, 200 µL of GB Buffer and 200 µL of ethanol were added to the mixture, which was subsequently loaded onto the spin column CB3. During centrifugation at 13,400 g for 30 s, the DNA bound to the silica–gel membrane. Subsequently, the DNA was washed with 500 µL of GD and 600 µL of PW washing buffers. Finally, the purified DNA was eluted using 50 µL of TE elution buffer. To assess the quality and concentration of the extracted DNA, a BioPhotometer D30 (Eppendorf, Hamburg, Germany) spectrophotometer was employed, which measured the absorbance of all samples at 260 nm (A260) and 280 nm (A280). Based on these measurements, a standard working concentration of 20 ng/µL was prepared for each specimen.

### 3.3. PCR Amplification and Sequencing

The PCR reaction mixture had a final volume of 20 µL and contained 2 µL of a 10x reaction buffer (with 15 mM MgCl_2_) (BioFlux, Hangzhou, China), 2.5 mM of each dNTP mixture (BioFlux, Hangzhou, China), 0.25–0.5 µM of each primer (for Salmonidae and *S. salar* species) in the duplex system, 0.2 µM of DNA barcoding primer in the single system (Table 1), 1 U BioReady rTaq DNA Polymerase (BioFlux, Hangzhou, China), and 20 ng DNA template. The amplification was performed in a Gentier 96E real-time PCR system (Tianlong, Xi’an, China).

For single PCR on each target species, the amplification was performed using the following temperature programme: 95 °C for 10 min; 40 cycles of 95 °C for 30 s, 60 °C for 30 s, and 72 °C for 40 s, with a final extension at 72 °C for 5 min. For DNA barcoding analysis, the amplification was performed using the following temperature programme: 94 °C for 2 min; 35 cycles of 94 °C for 30 s, 55 °C for 40 s, and 72 °C for 1 min, with a final extension at 72 °C for 10 min.

All the PCR products were checked on a 1.8% agarose gel (Biowest, Shanghai, China) stained with GelRed™ Nucleic Acid Gel Stain (Biotium, Hayward, CA, USA), and the presence of fragments of the expected length (Table 1) was assessed by a comparison with the standard DNA Marker DL100 (Sangon, Shanghai, China). The samples that contained the expected amplicon for the commercial products were sent to the company Sangon (Shanghai, China) for purification and bidirectional Sanger sequencing using the ABI 3730 DNA sequencer (Applied Biosystems Division, Foster City, CA, USA) (Table 2).

The obtained sequences were visualized, aligned, and edited using Clustal W in BioEdit software version 7.0.9 [36]. Fine adjustments were manually made after visual inspection. The generated DNA barcoding sequences were analyzed using the Identification System (IDs) on BOLD (Barcode of Life Data Systems) (http://www.boldsystems.org/index.php/IDS_OpenIdEngine, accessed on 14 August 2024). The correct assignment of individual samples to species was performed by calculating the expected similarity value (≥98%) [37] of reference sequence identity.

### 3.4. Duplex Real-Time PCR Coupled with Melting Curve Analysis

For the duplex real-time PCR, the reaction mixture was prepared in a final volume of 20 µL, comprising 10 µL of 2x SGExcel UltraSYBR Mixture (Sangon, Shanghai, China), 0.25 μM of each primer (Table 1), and 20 ng DNA template. The real-time PCR runs were executed on a fluorometric thermal cycler, the Gentier 96E real-time PCR system (TianLong, Xi’an, China), under the following thermal cycling conditions: 95 °C for 10 min, followed by 40 cycles of 95 °C for 30 s, and 60 °C for 30 s. Fluorescence signals were captured at the conclusion of each cycle on the SYBR Green channel. The Cq value (or takeoff point) was determined using its comparative quantification feature for the amplification data. Upon completion of amplification, the melting curve analysis was conducted by cooling the amplification products at 65 °C for 90 s, followed by a gradual heating from 65 °C to 95 °C at a rate of 0.5 °C/5 s. All samples were analyzed in duplicate to ensure reproducibility.

The specificity of the newly established duplex real-time PCR assay was evaluated using DNA from the nontarget species listed in Section 2.1 (Sample collection). The sensitivity of the real-time duplex PCR assay was assessed using a 5-fold serial dilution of the DNA template (from 20 to 0.032 ng). Moreover, experimental mixtures with different quantities of *O. masou* and *S. salar* tissue were also prepared. Progressive blending was simulated by adding a crescent proportion of *O. masou* and *S. salar* tissue from 0 to 100%.

## 4. Conclusions

This study combined duplex PCR with melting curve analysis to simultaneously detect *S. salar* and Salmonidae in a single PCR tube, which saved time and reduced the cost and amount of materials. Moreover, this novel assay holds the potential to bolster consumer confidence and ease regulatory adherence by ensuring the authenticity of products labeled as *S. salar* or products that contain Salmonidae ingredients. Moreover, the versatility and scalability of this method render it suitable for use in diverse settings, ranging from research laboratories to large-scale industrial processing facilities, emphasizing its significance as a valuable asset for the seafood industry.

## Figures and Tables

**Table 1:** Primers used in the present study.

Target Species	Target Gene	Primer	Sequence (5′→3′)	Amplicon (bp)	Reference
-	Cytochrome c oxidase subunit I (COI)	mlCOIintF (F)	GGWACWGGWTGAACWGTWTAYCCY CC	313	[31]
		jgHCO2198 (R)	TAIACYTCIGGRTGICCRAARAAYCA		
Salmonidae	Cytochrome b (Cyt b)	FCYTB346	CGGAGTTGTACTTCTACTTCTCAC	159	[19]
		RCYTB530	AAGTGGAAGGCGAAAAATCGT		
Atlantic salmon (*S. salar*)	COI	SalmonF-1	CCTCCATTTGGCTGGTATTTCT	220	[32]
		SalmonR-1	GAGGGAGAGTAACAAAAGGACG		

**Table 2:** Method application on commercial products.

Sample Code	Processed Method	Duplex Real-Time PCR			DNA Barcoding (Similarity ≥ 98%)
		Cq	*S. salar*	Salmonidae	
s1	Frozen products	17.6	+	+	*S. salar*
s2		15.2	+	+	*S. salar*
s3		15.8	+	+	*S. salar*
s4		18.8	+	+	*S. salar*
s5		15.5	+	+	*S. salar*
s6		17.6	−	+	*O. mykiss*
s7		15.2	−	+	*O. mykiss*
s8		15.5	+	+	*S. salar*
s9		15.0	+	+	*S. salar*
s10		17.4	+	+	*S. salar*
s11		15.6	+	+	*S. salar*
s12		15.0	+	+	*S. salar*
s13		20.6	−	+	*O. keta*
s14	Dried products	-	−	−	−
s15		21.3	+	+	*S. salar*
s16		20.4	−	+	*O. gorbuscha*
s17		-	−	−	−
s18		-	−	−	−
s19		-	−	−	−
s20		22.5	+	+	*S. salar*

Note: + represents the presence of melting peak. − represents the absence of melting peak, or the failure of DNA barcoding assay. Words with grey background are the dried products. For duplex real-time PCR, the Cq value was added.

## Data Availability

Data were contained within the article. The data presented in this study are available.

## Supplementary Materials

The following supporting information can be downloaded at https://www.mdpi.com/article/10.3390/molecules29204904/s1. Figure S1: Single real-time PCR for specificity test.
