# Supplementary material for: A Novel Method for Rapid Screening of Salmonidae Ingredients and Accurate Detection of Atlantic Salmon (Salmo salar) Simultaneously Using Duplex Real-Time PCR Coupled with Melting Curve Analysis
Source: Molecules. 2024 Oct 16;29(20):4904. doi: 10.3390/molecules29204904 (PMC11510278; doi:10.3390/molecules29204904)
Supplement: Supplementary file 1 [file molecules-29-04904-s001.zip › Figure S1 Simplex real-time PCR for specificity test.pdf]

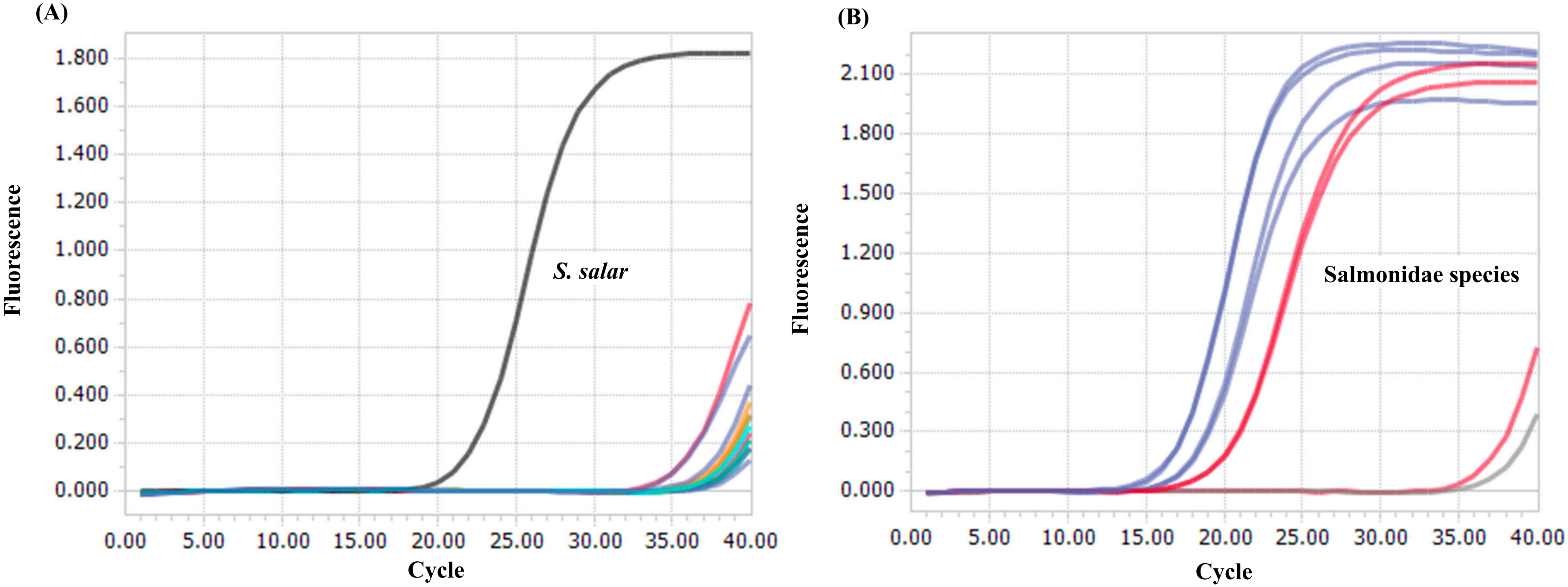

Figure S1 Single real-time PCR for specificity test

(A) *S. salar* species-specific primer; **(B)** Salmonidae-specific primer

The reaction was also performed in 20 μL containing similar PCR reagents to the ones described in section **2.3 PCR amplification and sequencing**. While the amplification was made on a LightCycler® 96 real time PCR system (Roche, Basel, Switzerland). The used species included: *O. mykiss, O. keta, O. masou, O. nerka, O. gorbuscha, O. tshawytscha, S. salar, Gadus chalcogrammus, Melanogrammus aeglefinus, Lateolabrax japonicus, Epinephelus costae, Pennahia argentata, Upeneus japonicas, Mullus barbatus, Lates niloticus, Xiphias gladius, Sardina pilchardus, Polydactylus sextarius, Odontobutis potamophila, Anoplopoma fimbria, Thunnus obesus, Pleuronectes platessa, Cyprinus carpio, and Thunnus albacores.*
